# Experimental study of the nonreciprocal effective interactions between microparticles in an anisotropic plasma

**DOI:** 10.1038/s41598-020-70441-z

**Published:** 2020-08-12

**Authors:** E. A. Lisin, O. F. Petrov, E. A. Sametov, O. S. Vaulina, K. B. Statsenko, M. M. Vasiliev, J. Carmona-Reyes, T. W. Hyde

**Affiliations:** 1 Joint Institute for High Temperatures, Russian Academy of Sciences, Moscow, Russia 125412; 2 Moscow Institute of Physics and Technology, Dolgoprudny, Russia 141700; 3 Center for Astrophysics, Space Physics and Engineering Research (CASPER), Baylor University, Waco, TX 76798-7310 USA

**Keywords:** Self-assembly, Plasma physics, Statistical physics, Characterization and analytical techniques

## Abstract

There is a variety of cases in nature when the action–reaction symmetry is broken. In particular, suitable conditions for this are realized in colloidal suspensions and complex plasmas. Since the first theories and simulations of the nonreciprocal effective interactions between microparticles in complex plasmas were published in 1995–1996, there have been hundreds of studies in the theoretical development of this theme. However, despite such a rich theoretical background, one of the important unsolved problems is a direct experimental determination of the nonreciprocal interparticle interaction forces. Here, we studied experimentally in detail the forces of the nonreciprocal effective interaction between microparticles suspended a radio-frequency produced plasma sheath. For this purpose, an experimental method based on an analysis of the spectral density of random processes in an open dissipative two-particle system was developed. In contrast to previous investigations, the proposed method takes into account random and dissipative processes in the system, does not require a special design of the experimental setup and any external perturbations, pre-measurements of external fields and any assumptions about the type of interaction. We found that even small charge changes of one particle, caused by its thermal motion in a wake field of another particle, can lead to a significant change in the effective (measurable) interaction between the particles.

## Introduction

Recently, quite a few studies have appeared on the so-called “violation” of the interaction symmetry. Such a formal failure to comply with Newton's third law may arise, for example, when a particle subsystem in a medium is considered while the medium itself is indirectly taken into account through the potential of interparticle interaction, dissipative forces or as a source of particle kinetic energy. A striking example of such systems are some types of soft matter e.g., flowing colloidal suspensions^1–6^, active colloids^7–11^ and gas-discharge complex (dusty) plasmas^12–23^ where the geometry of interactions between particles plays a key role in the processes of self-organization, self-assembly, transfer and redistribution of energy, and nonequilibrium phase transitions. In addition to the fundamental physics that can be examined, the study of these systems is also of particular interest for nano- and micro-technological applications^24–30^.

The “non-fulfillment” of Newton's third law may arise due to different properties of dispersed phase particles suspended in a medium. For example, in active colloids, it happens due to different chemical reactions taking place on the surface of active colloidal particles in a solution^28–30^. As a result, a particle that is in a non-uniform chemical field created by a second particle will be affected by a diffusiophoretic force due to a chemical concentration gradient^7,8^. If the particles themselves are of a different sort or, being identical, have a chemically bifunctional surface (i.e., Janus particles), then the symmetry of this interaction type between them will be broken. In dusty plasmas, the difference in the dielectric constant of charged spherical macroparticle in an equilibrium isotropic plasma leads to a nonreciprocal effective electrostatic interaction between them due to the different angular dependence of the space charge distribution near the surface of particles^31^. The interaction between charged dust particles in an isotropic plasma can be also provided by forces of a nonelectrical nature, which are associated with so-called shadowing (i.e., scattering or absorption) of the ion or neutral gas fluxes towards one dust particle by another one^32–34^. For the particles of different sizes, the shadow interaction becomes nonreciprocal^35^.

Non-reciprocity of interparticle interaction forces may also occur due to external forces causing flows of the medium surrounding the particles. For example, in colloidal suspensions, the flow of a colloidal solution of relatively closely spaced big colloidal particles can also lead to a violation of the interaction symmetry due to depletion or entropic forces^1,3–5^. In dusty plasmas, a strong electric field near the electrode leads to ion drift^36^. When micron-sized particles are in a plasma with an ion flux, they acquire a significant negative charge (10^3^–10^4^ elementary charges) due to high electron mobility and can create a disturbed, positive spatial region (wake ion trail) in the ion flow direction^37,38^. In this case, negatively charged dust particles experience both electrostatic repulsion from similarly charged neighboring particles as well as an effective attraction to the positive space charge arising in their wake.

Since the first theories and simulations of the wakes in complex plasmas were published by Vladimirov et al.^39^ and Schweigert et al.^40^, there have been hundreds of studies in the theoretical development of this theme. However, despite such a rich theoretical background, one of the important unsolved problems in complex plasmas is a direct experimental determination of the wake-mediated interparticle interaction forces.

The first attempts to analyze the wake-mediated interaction forces were undertaken by Takahashi et al.^41^ and Meltzer et al.^42^. By evaluating the dynamic responses of microparticles to laser manipulations it was shown that the behavior of particles in such systems is non-reciprocal. Later, Hebner et al.^43^ using a gravity-driven dynamic probe and theoretical models of the electric fields and external forces acting on a system of dust particles, estimated the horizontal projection (perpendicular to the ion flow direction) of the attraction force of the wake on the dust particle.

Jung et al. attempted to study the longitudinal structure of the ion wake behind a dust particle using an approaching second particle as a probe^44^. However, the authors concluded that for this case, an ion focus was formed not behind the dust particle, but only behind the probe.

Recently, Qiao et al.^45^ proposed a non-perturbative method for determining the derivatives of the interaction forces, based on the scanning mode spectra (SMS) analysis of the particles’ thermal motion. Unfortunately, this method does not take into account dissipative and random processes, which can result in significant measurement errors in the presence of non-reciprocity, even in a weakly dissipative system^46^. However, it is much more significant that in both Refs.^44,45^ the charge of the test particle is considered constant. As will be shown, this simplification can lead to large measurement errors.

This paper examines the forces of the nonreciprocal effective interaction between microparticles in a radio-frequency (RF) produced plasma sheath, depending on the buffer gas pressure and the discharge power, which determine the drift of the ion component and the structure of the wake-field. For this purpose, we use an experimental method based on an analysis of the spectral density of random processes. In contrast to previous investigations, our method does not require a special design of the experimental setup or any external perturbations of a system, pre-measurements of external fields or assumptions about the type of interaction.

## Experimental setup

**Figure 1:**
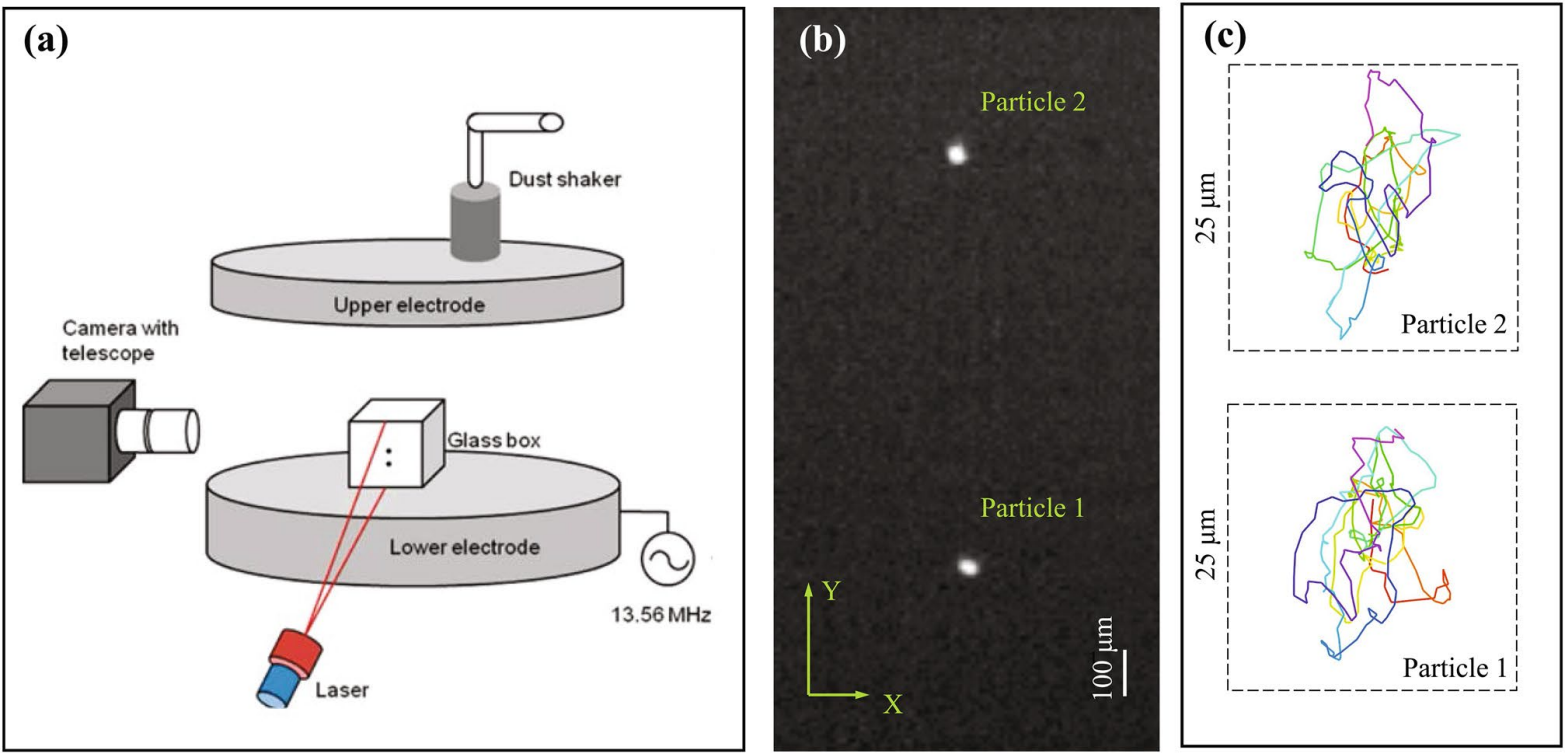
(**a**) Sketch of the experimental setup with a RF discharge. A 12.7 mm × 12.7 mm × 12.7 mm glass box placed on the lower electrode creates the electric potential needed to confine the microparticles. (**b**) The video frame with the image of the particles levitating in the glass box above the lower electrode under a pressure of 136 mTorr and RF power of 2.33 W. (**c**) The trajectories of the particles obtained as a result of computer processing of the video record of their movement for 1 s.

The experiments were carried out in a Gaseous Electronics Conference (GEC) RF reference cell having two 8 cm-diameter electrodes separated by a distance of 2.54 cm. The lower electrode was powered at 13.56 MHz while the upper ring-shaped electrode and chamber acted as ground. A 12.7 mm × 12.7 mm × 12.7 mm (height × length × width) glass box was placed on the lower powered electrode to create the electric potential needed to confine the dust particles (see Fig. 1a). Experiments were conducted in Argon plasma at 70, 136 and 280 mTorr employing rf powers of 1.9–14.3 W. Pair interparticle interactions were examined since they are well suited for study using a two-particle system, due to the fact that a consideration of collective effects is not required. To properly explore the ion wake, which is formed behind the particles downstream of the ion flow, the particles should be placed one below the other. In such a configuration, the lower particle represents a probe for measuring the wake-mediated force from the upper particle. In this case, a two-particle pair of 8.89 μm melamine formaldehyde (MF) particles was used to form a vertical chain within the glass box. All other particles were removed from the system by decreasing the rf power until only a two-particle vertical pair was left^47,48^. A vertically fanned laser sheet illuminated the particles and side view images were recorded for 50 s using a HD camera at 250 fps. An image of the particles levitating in the glass box is shown in Fig. 1b. The resulting series of images was analyzed to obtain each particle’s trajectory. A representative example of the particle trajectories obtained as a result of computer processing of the video of their motion are shown in Fig. 1c.

## Results and discussion

The object of study can be considered as a stationary system of two interacting particles with indices 1 and 2 immersed in a dissipative medium and located in an external non-uniform force field ${\mathbf{F}}_{{{\text{ext}}}}$. We assume that the particles have equal masses $M$ and a spherical shape with the same radius. Therefore, they have the same coefficient of friction in a medium $\nu$. We also take into account that the interparticle interaction symmetry can be broken, i.e. ${\mathbf{F}}_{kj} \ne {\mathbf{F}}_{jk}$, where ${\mathbf{F}}_{kj}$ is the force exerted by the particle $k$ on the particle $j$. Hereinafter $j,k = 1 \ldots 2$ and $j \ne k$.

For small deviations $\delta_{j}^{{({\upxi })}}$ from the equilibrium positions compared to the equilibrium interparticle distance, the forces acting on the particle $j$ in ξ-direction can be linearized: $F_{kj}^{(\upxi )} = \left\langle {F_{kj}^{(\upxi )} } \right\rangle + \left( {\delta_{j}^{(\upxi )} - \delta_{k}^{(\upxi )} } \right) \, Mf_{kj}^{(\upxi )}$, $F_{ext, \, j}^{(\upxi )} = \left\langle {F_{ext, \, j}^{(\upxi )} } \right\rangle + \delta_{j}^{(\upxi )} \, Mf_{j}^{(\upxi )}$, and the particles can be treated as nonreciprocal coupled harmonic oscillators, driven by random processes $b_{j}^{{({\upxi })}} (t)$:

1 $$\ddot{\delta }_{j}^{{({\upxi })}} = - \nu \dot{\delta }_{j}^{{({\upxi })}} + \left( {\delta_{j}^{{({\upxi })}} - \delta_{k}^{{({\upxi })}} } \right)f_{kj}^{{({\upxi })}} - \delta_{j}^{{({\upxi })}} f_{j}^{{({\upxi })}} + b_{j}^{{({\upxi })}} (t)$$

where $f_{kj}^{(\upxi )}$ and $f_{j}^{(\upxi )}$ are the directional derivatives of the ξ-components of the interaction specific force ${\mathbf{F}}_{kj} /M$ and external specific force ${\mathbf{F}}_{{{\text{ext}}}} /M$ and the ${\upxi }$ superscript denotes the selected Cartesian coordinate axis (${\upxi } \equiv {\text{X}}$ or ${\text{Y}}$). The particles are considered to be strongly coupled thus they are in thermal motion near their equilibrium positions.

The spectral densities $G_{j}^{(\upxi )} \left( \omega \right)$ of the particle oscillations $\delta_{j}^{{({\upxi })}}$ as well as the spectral densities $G_{ + }^{{({\upxi })}} \left( {\upomega } \right)$ and $G_{ - }^{{({\upxi })}} \left( {\upomega } \right)$ for $\delta_{ + }^{(\upxi )} = \delta_{j}^{(\upxi )} + \delta_{k}^{(\upxi )}$ and $\delta_{ - }^{(\upxi )} = \delta_{j}^{(\upxi )} - \delta_{k}^{(\upxi )}$, respectively, are completely determined by the parameters $f_{kj}^{(\upxi )}$, $f_{j}^{(\upxi )}$, $\nu$ and the effective temperatures $T_{j}^{{({\upxi })}}$ of the thermostat-like regulating stochastic processes $b_{j}^{{({\upxi })}} (t)$, see “Methods”. Here we assume that in general $T_{j}^{{({\upxi })}} \ne T_{k}^{{({\upxi })}}$ and $T_{j}^{{({\text{X}})}} \ne T_{j}^{{({\text{Y}})}}$ taking into account the inhomogeneity and anisotropy of the plasma sheath.

**Figure 2:**
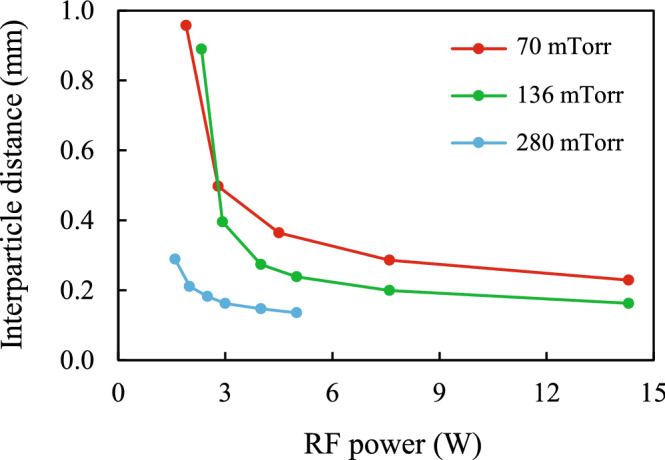
Dependence of the mean interparticle distance $\Delta$ on the RF power at the gas pressures shown.

Figure 2 shows the dependence of the mean distance between particles $\Delta$ at a discharge power *W* and various gas pressures *P*. With increasing power and pressure, the distance $\Delta$ decreased from 0.96 mm at *W* = 1.9 W and *P* = 70 mTorr to 0.16 mm at *W* = 5 W and *P* = 280 mTorr. In all experiments the standard deviation $\sigma_{\Delta }$ from the value of $\Delta$ was in the range of $0.015\Delta$ to $0.02\Delta$.

**Figure 3:**
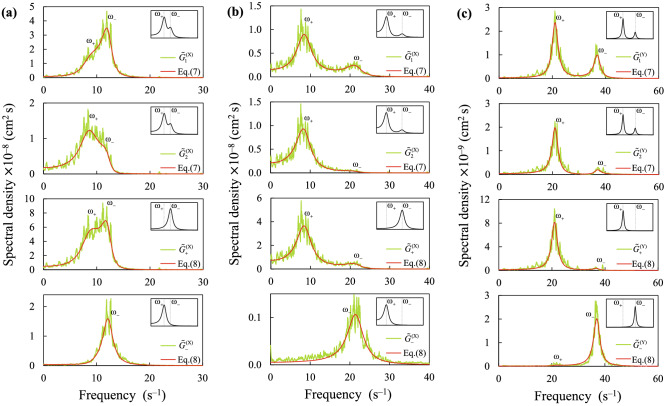
Spectral densities $\tilde{G}_{1}^{{{{(\upxi )}}}}$, $\tilde{G}_{2}^{{{{(\upxi )}}}}$, $\tilde{G}_{ + }^{{{{(\upxi )}}}}$ and $\tilde{G}_{ - }^{{{{(\upxi )}}}}$ of the oscillations of particles (**a**) in the horizontal direction (${\upxi } \equiv {\text{X}}$) for a gas pressure *P* of 136 mTorr and a discharge power *W* of 4 W; (**b**) – ${\upxi } \equiv {\text{X}}$, *P* = 136 mTorr, *W* = 14.3 W; (**c**) in the vertical direction (${\upxi } \equiv {\text{Y}}$) at *P* = 70 mTorr, *W* = 14.3 W; and their analytical approximations by Eqs. (7) and (8) from “Methods”. For comparison, schematic distributions of the corresponding spectral densities for particles interacting with a purely repulsive spherically symmetric potential are shown in the inserts. Spectral peaks correspond to oscillations at characteristic frequencies ${\upomega }_{ - }$ and ${\upomega }_{ + }$. (Note that the characteristic frequencies coincide with the eigen frequencies of the system at zero friction.)

The particle trajectories obtained in the experiments were processed using a fast Fourier transform. As a result, for each pair of values of pressure and power, spectral densities of oscillations of the particle coordinates ($\tilde{G}_{1}^{{{{(\upxi )}}}}$ and $\tilde{G}_{2}^{{{{(\upxi )}}}}$) and their sums ($\tilde{G}_{ + }^{{{{(\upxi )}}}}$), as well as relative displacements of the particles ($\tilde{G}_{ - }^{{{{(\upxi )}}}}$) were obtained. Hereinafter, the lower particle will have the index 1, and the upper tilde will mean that the value is measured in the experiment. Also for definiteness, the Y axis is directed vertically (opposite to gravity), as shown in Fig. 1b. As a representative example, Fig. 3 shows the spectral densities ($\tilde{G}_{1}^{{{{(\upxi )}}}}$, $\tilde{G}_{2}^{{{{(\upxi )}}}}$, $\tilde{G}_{ + }^{{{{(\upxi )}}}}$ and $\tilde{G}_{ - }^{{{{(\upxi )}}}}$), obtained in the experiment at pressures of 70 and 136 mTorr and discharge powers of 4 and 14.3 W. For comparison, Fig. 3 also includes schematic distributions of the corresponding spectral densities for particles interacting with a purely repulsive spherically symmetric potential. Spectral peaks correspond to oscillations at characteristic frequencies ${\upomega }_{ - }$ and ${\upomega }_{ + }$. Note that the characteristic frequencies coincide with the eigen frequencies of the system at $\nu \to 0$. A weak peak at the low characteristic frequency ${\upomega }_{ + }$ in the spectra of relative displacements $\tilde{G}_{ - }^{{\text{(Y)}}}$ along the vertical axis (see Fig. 3c) may occur due to the slight difference in the gradients of the external forces acting on the particles. The oscillation amplitudes at the high characteristic frequency ${\upomega }_{ - }$ of the lower and upper particles are significantly different (see the spectra $\tilde{G}_{1}^{{{{(\upxi )}}}}$ and $\tilde{G}_{2}^{{{{(\upxi )}}}}$). This is a sign of the inequality of the derivative forces with which the particles act on each other. The presence of a peak at the frequency ${\upomega }_{ + }$ in the spectrum $\tilde{G}_{ + }^{{\text{(X)}}}$, as well as a pronounced peak at the frequency ${\upomega }_{ - }$ in the spectrum $\tilde{G}_{ - }^{{\text{(X)}}}$(see Figs. 3a,b) indicates an effective attraction of the lower particle to the upper. Figure 3 also contains graphs of approximating functions (see Eqs. (7) and (8) in “Methods”), dependent on the parameters $f_{21}^{{({\upxi })}}$, $f_{12}^{{({\upxi })}}$, $f_{1}^{{({\upxi })}}$, $f_{2}^{{({\upxi })}}$, $T_{1}^{{({\upxi })}}$, $T_{2}^{{({\upxi })}}$ and $\nu$ that provide the best agreement between the analytical curves and all experimentally measured spectral densities ($\tilde{G}_{1}^{{{{(\upxi )}}}}$, $\tilde{G}_{2}^{{{{(\upxi )}}}}$, $\tilde{G}_{ + }^{{{{(\upxi )}}}}$ and $\tilde{G}_{ - }^{{{{(\upxi )}}}}$).

The normalized friction coefficient ${\nu \mathord{\left/ {\vphantom {\nu P}} \right. \kern-\nulldelimiterspace} P}$ obtained from the spectra is independent of pressure and discharge power and is equal to $1.38 \pm 0.08$ s^–1^/Pa. An estimate made by the Epstein formula^49^ for the diffuse reflection of the gas atoms on the particle surface^44^ gives $\nu /P$ = $1.37$ s^–1^/Pa.

**Figure 4:**
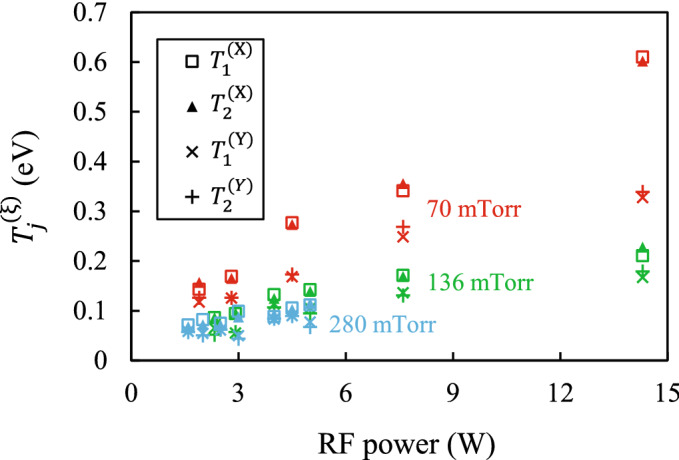
Effective temperatures $T_{j}^{{({\upxi })}}$ of the stochastic processes driving the microparticles, depending on the RF power at different gas pressures.

Figure 4 shows the effective temperatures $T_{j}^{{({\upxi })}}$ of the stochastic processes driving the microparticles, depending on RF power *W* at different gas pressures *P*. In all experiments the effective temperatures $T_{j}^{{({\upxi })}}$ increases with increasing RF power and decreases with increasing gas pressure. The resulting pressure dependence is in good qualitative agreement with previous temperature measurements of a single dust particle in a radio-frequency produced plasma sheath^50^. Across all discharge parameters, the effective temperatures $T_{j}^{{({\text{X}})}}$ of the stochastic processes driving the microparticles in the horizontal direction exceed the corresponding temperatures $T_{j}^{{({\text{Y}})}}$ for the vertical direction. This fact is also consistent with previous studies^50^. Figure 4 also shows that the temperatures corresponding to the stochastic processes acting on the lower and upper particles are approximately equal.

**Figure 5:**
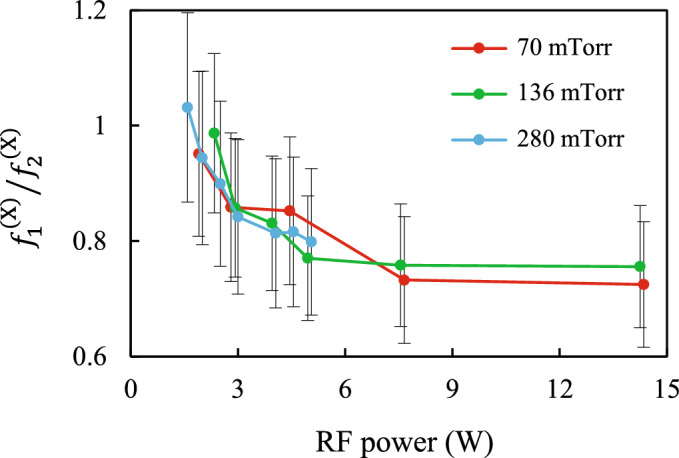
Ratio ${{f_{1}^{{({\text{X}})}} } \mathord{\left/ {\vphantom {{f_{1}^{{({\text{X}})}} } {f_{2}^{{({\text{X}})}} }}} \right. \kern-\nulldelimiterspace} {f_{2}^{{({\text{X}})}} }}$ of the derivatives of the external specific forces which confine the particles in the horizontal direction, depending on the RF power at different gas pressures.

Figure 5 shows the ratio ${{f_{1}^{{({\text{X}})}} } \mathord{\left/ {\vphantom {{f_{1}^{{({\text{X}})}} } {f_{2}^{{({\text{X}})}} }}} \right. \kern-\nulldelimiterspace} {f_{2}^{{({\text{X}})}} }}$ of the derivatives of the external specific forces which confine the particles in the horizontal direction, depending on the discharge power *W* at different gas pressures *P*. Taking into account the measurement error, we see that for all *P* the ratio ${{f_{1}^{{({\text{X}})}} } \mathord{\left/ {\vphantom {{f_{1}^{{({\text{X}})}} } {f_{2}^{{({\text{X}})}} }}} \right. \kern-\nulldelimiterspace} {f_{2}^{{({\text{X}})}} }}$ decreases from ~ 1 to ~ 0.8 with increasing W. Earlier Carstensen et al.^51^ showed experimentally that the position dependence of the charge on a solitary particle in an rf discharge is negligible when its position (height) relative to the bottom electrode is changed by 0.4 mm or less, which is comparable to the characteristic average interparticle distance $\Delta$ in our experiments. In this case, the gradient of the electric field of the undisturbed plasma sheath in the area of levitation of particles can be considered to be a constant value where the value $f_{j}^{{({\upxi })}}$ is proportional to the charge-to-mass ratio of the particle ${{Q_{j} } \mathord{\left/ {\vphantom {{Q_{j} } M}} \right. \kern-\nulldelimiterspace} M}$. Since we used the monodisperse particles, we can write ${{f_{1}^{{({\text{X}})}} } \mathord{\left/ {\vphantom {{f_{1}^{{({\text{X}})}} } {f_{2}^{{({\text{X}})}} }}} \right. \kern-\nulldelimiterspace} {f_{2}^{{({\text{X}})}} }} \approx {{Q_{1} } \mathord{\left/ {\vphantom {{Q_{1} } {Q_{2} }}} \right. \kern-\nulldelimiterspace} {Q_{2} }}$. Consequently, at higher discharge powers, we observe a decrease in the charge of the lower particle by ~ 25%. This result is in agreement with previous experiments^45,51^, as well as with calculations^52^. This charge reduction is due to the ion wake and was predicted by Vladimirov et al.^53^. As the discharge power increases, the speed of the ion drift increases, and the upstream particle amplifies the ion flows to the lower particle. For a minimum discharge power, the ratio ${{f_{1}^{{({\text{X}})}} } \mathord{\left/ {\vphantom {{f_{1}^{{({\text{X}})}} } {f_{2}^{{({\text{X}})}} }}} \right. \kern-\nulldelimiterspace} {f_{2}^{{({\text{X}})}} }}$ is close to one, although taking into account the permissible error, it can be several percent higher than one. This may also be due to a small difference in the masses of the particles.

**Figure 6:**
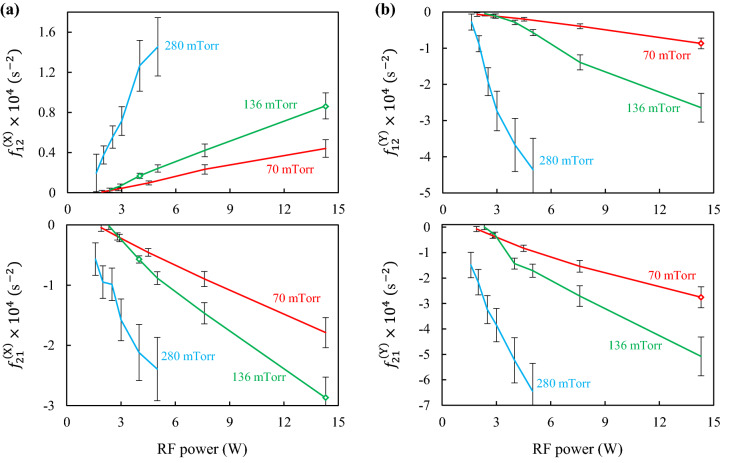
Derivatives of the specific interaction forces $f_{21}^{{({\upxi })}}$ and $f_{12}^{{({\upxi })}}$ obtained at different pressures and RF powers in the X direction (**a**) and in the Y direction (**b**). Diamonds denote the experiments, for which the spectral densities are presented in Fig. 3.

**Figure 7:**
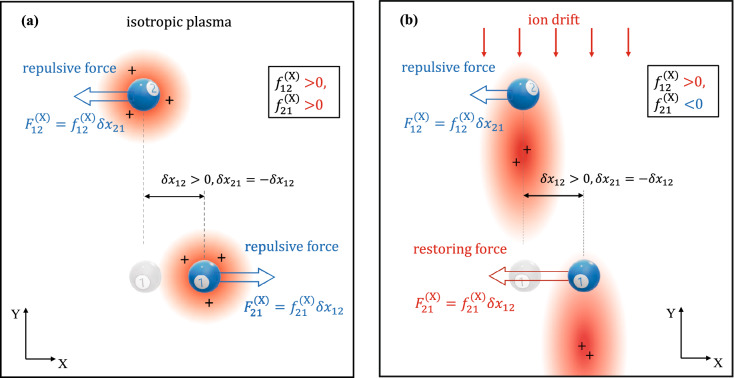
Forces acting horizontally between particles in an isotropic plasma (**a**) and when a field is applied (**b**) and the nonreciprocity is rather strong $\left( {{{F_{21}^{{({\text{X}})}} } \mathord{\left/ {\vphantom {{F_{21}^{{({\text{X}})}} } {F_{12}^{{({\text{X}})}} }}} \right. \kern-\nulldelimiterspace} {F_{12}^{{({\text{X}})}} }} < - 1} \right)$.

The derivatives of the specific interaction forces $f_{21}^{{({\upxi })}}$ and $f_{12}^{{({\upxi })}}$ obtained at different pressures ($P$ = 70, 136 and 280 mTorr) and discharge powers $W$(in the range from 1.6 to 14.3 W) are shown in Fig. 6. We see that the absolute values of $f_{21}^{{({\upxi })}}$ and $f_{12}^{{({\upxi })}}$ increase with increasing power and pressure (with exception of the points *P* = 70 and 136 mTorr with $W < 3$ W). In the X direction these derivatives have opposite signs at all pressures and discharge powers: $f_{21}^{{({\text{X}})}} < 0$ and $f_{12}^{{({\text{X}})}} > 0$. Negative values $f_{21}^{{({\text{X}})}}$ indicate that with a horizontal displacement of the lower particle from its equilibrium position, an effective attracting force arises from the upper particle and tends to return the lower particle to the equilibrium position. For comparison, we note that if the particles interacted through a purely repulsive spherically symmetric potential, then both derivatives $f_{21}^{{({\text{X}})}}$ and $f_{12}^{{({\text{X}})}}$ would be positive and equal to each other (see Fig. 7a). According to popular wake-field models, such as the point-wake model^14,16,17,21,40^ and the Kompaneets model^54^, for the case where there is an ion drift weakly disturbed by the upstream microparticle, the derivatives $f_{21}^{(X)}$ and $f_{12}^{{({\text{X}})}}$ may not be equal (due to violation of the interaction symmetry) while still positive. However, with an increase in the intensity (amplitude) of the wake field, the derivative $f_{21}^{{({\text{X}})}}$ may change sign due to the strong effective attraction of the lower negatively charged microparticle to the positive volume charge of the ionic trace (see Fig. 7b). We believe that this effect has been observed in our experiments.

The results of measuring the derivatives of the specific interaction forces in the Y direction turned out to be quite unexpected. Theoretical models^21,40,54^ predict that in the presence of a weak wake field, the value of the derivative $f_{21}^{{({\text{Y}})}}$ will be less than $f_{12}^{{({\text{Y}})}}$, i.e. $f_{21}^{{({\text{Y}})}} < 0$, $f_{12}^{{({\text{Y}})}} < 0$, $\left| {f_{21}^{{({\text{Y}})}} } \right| < \left| {f_{12}^{{({\text{Y}})}} } \right|$. As the amplitude of the attractive part of the wake field increases, the derivative $f_{21}^{{({\text{Y}})}}$ may change sign and become positive although this does not necessarily depend on the position of the maximum of the ion wake (i.e., between particles or behind both). However, in our experiments, for all values of *P* and *W* (except for *P* = 136 mTorr, *W* = 2.33 W), we found $f_{21}^{{({\text{Y}})}} < 0$, $f_{12}^{{({\text{Y}})}} < 0$ and $\left| {f_{21}^{{({\text{Y}})}} } \right| > \left| {f_{12}^{{({\text{Y}})}} } \right|$.

To explain the results of these experiments, it is necessary to take into account that the charge on the lower particle $Q_{1}$ is a function of the position of the lower particle relative to the upper one:

2 $$Q_{1} \, = \, \left\langle {Q_{1} } \right\rangle \left[ {1 + \left( {\delta_{2}^{{({\upxi })}} - \delta_{1}^{{({\upxi })}} } \right)q^{\prime}_{1} } \right],$$

where $q^{\prime}_{1}$ is the charge gradient normalized to the equilibrium charge $\left\langle {Q_{1} } \right\rangle$. According to Ref.^51^, we neglect the dependence of the charge of each of the particles on the height from the lower electrode although this value may fluctuate due to thermal displacements of both particles. In this case, in the equation of motion (1) the derivative $f_{21}^{{({\upxi })}}$ should be replaced by the effective derivative

3 $$f_{21,eff}^{{({\upxi })}} = f_{21}^{{({\upxi })}} - q^{\prime}_{1} {{\left\langle {F_{{{\text{ext, }}1}}^{{{{(\upxi )}}}} } \right\rangle } \mathord{\left/ {\vphantom {{\left\langle {F_{{{\text{ext, }}1}}^{{{{(\upxi )}}}} } \right\rangle } M}} \right. \kern-\nulldelimiterspace} M},$$

where the motion of particle 2 is still described by Eq. (1) without changes, and the derivatives $f_{21}^{{({\upxi })}}$ and $f_{12}^{{({\upxi })}}$ now include an additional term depending on $q^{\prime}_{1}$. Since $\left\langle {F_{{{\text{ext, }}1}}^{{\text{(X)}}} } \right\rangle = 0$, Eq. (3) is relevant only for displacements of the particles along the Y axis. As particle 1 approaches the potential maximum of the wake field generated by particle 2, the absolute value of the negative charge $Q_{1}$ will decrease. From balance of forces, we can write ${{{-}\left\langle {F_{{{\text{ext, }}1}}^{{\text{(Y)}}} } \right\rangle } \mathord{\left/ {\vphantom {{{-}\left\langle {F_{{{\text{ext, }}1}}^{{\text{(Y)}}} } \right\rangle } M}} \right. \kern-\nulldelimiterspace} M} \approx g = 980$ cm/c^2^, which implies that the quantity $q^{\prime}_{1} {{\left\langle {F_{{{\text{ext, }}1}}^{{\text{(Y)}}} } \right\rangle } \mathord{\left/ {\vphantom {{\left\langle {F_{{{\text{ext, }}1}}^{{\text{(Y)}}} } \right\rangle } M}} \right. \kern-\nulldelimiterspace} M}$ entering into Eq. (7) can be significant. In our experiments, in order for the value $q^{\prime}_{1} {{\left\langle {F_{{{\text{ext, }}1}}^{{\text{(Y)}}} } \right\rangle } \mathord{\left/ {\vphantom {{\left\langle {F_{{{\text{ext, }}1}}^{{\text{(Y)}}} } \right\rangle } M}} \right. \kern-\nulldelimiterspace} M}$ to be greater than or on the order of $f_{21}^{{({\text{Y}})}}$, it is sufficient that the charge fluctuation is 0.1–1% for the measured standard deviations $\sigma_{\Delta } \approx 0.02\Delta$ of the interparticle distance.

**Figure 8:**
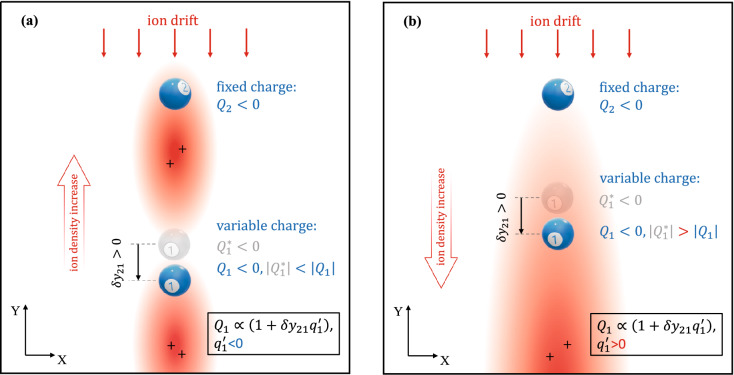
Changing the lower particle charge depending on its position relative to the ion wake of the upper particle: between particles (**a**) or behind both (**b**).

Although measurements of the derivative $f_{21}^{{({\text{Y}})}}$ are not suitable for analyzing real interaction between particles, they may provide useful information on the position of the ion wake relative to the particles. We introduce the parameter $d^{ * } = {d \mathord{\left/ {\vphantom {d \Delta }} \right. \kern-\nulldelimiterspace} \Delta }$, where $d$ is the distance from the particle to the potential maximum generated by its wake field. For $d^{ * } < 1$ we have $q^{\prime}_{1} < 0$, and if $d^{ * } > 1$ then $q^{\prime}_{1} > 0$ (see Fig. 8). According to the models^21,40,54^ and the conditions of stability^21,55^, for a strong nonreciprocity we also have $f_{21}^{{({\text{Y}})}} > 0$, $f_{12}^{{({\text{Y}})}} < 0$ and $\left| {f_{21}^{{({\text{Y}})}} } \right| < \left| {f_{12}^{{({\text{Y}})}} } \right|$. In this case, for an increase in the amplitude of oscillations of the charge magnitude $Q_{1}$, the effective derivative $f_{21,eff}^{{({\text{Y}})}}$ changes to a negative value at $d^{ * } < 1$, while for $d^{ * } > 1$ it is positive but greater than $\left| {f_{12}^{{({\text{Y}})}} } \right|$. Comparison of the derivatives of interaction forces at different positions of the maximum of the ion wake (i.e., between particles or behind both) and different models of the lower particle charge (fixed or variable) is given in Table 1. The calculations for Table 1 were performed for the point-wake model^21^ of the particle interaction with the rather strong nonreciprocity $\left( {{{f_{21}^{{({\text{Y}})}} } \mathord{\left/ {\vphantom {{f_{21}^{{({\text{Y}})}} } {f_{12}^{{({\text{Y}})}} }}} \right. \kern-\nulldelimiterspace} {f_{12}^{{({\text{Y}})}} }} < 0} \right)$ taking into account the conditions of the vertical stability^21,55^ and the charge fluctuation sufficient to satisfy the condition $\left| {{{f_{21,eff}^{{({\text{Y}})}} } \mathord{\left/ {\vphantom {{f_{21,eff}^{{({\text{Y}})}} } {f_{12}^{{({\text{Y}})}} }}} \right. \kern-\nulldelimiterspace} {f_{12}^{{({\text{Y}})}} }}} \right| > 1$. Thus, the fulfillment of condition

4 $${{f_{21,eff}^{{({\text{Y}})}} } \mathord{\left/ {\vphantom {{f_{21,eff}^{{({\text{Y}})}} } {f_{12}^{{({\text{Y}})}} }}} \right. \kern-\nulldelimiterspace} {f_{12}^{{({\text{Y}})}} }} > 1$$

may indicate that the attractor of the potential of interparticle interaction is located between the particles. Nevertheless, the opposite statement may be incorrect in some cases. Note that the proposed model of the fluctuating charge of the lower particle allows us to explain the anomalously high ratio $$\left| {{{f_{21,eff}^{{({\text{Y}})}} } \mathord{\left/ {\vphantom {{f_{21,eff}^{{({\text{Y}})}} } {f_{12}^{{({\text{Y}})}} }}} \right. \kern-\nulldelimiterspace} {f_{12}^{{({\text{Y}})}} }}} \right|$$$$\gg$$ 1 observed in Ref.^44^.

**Table 1:** Comparison of the derivatives of interaction forces at different positions of the maximum of the ion wake (i.e., between particles or behind both) and different models of the lower particle charge (fixed or variable).

Position of the ion wake	Between particles		Behind both		No wakes (isotropic plasma)
Fixed/variable charge ${Q}_{1}$	Fixed	Variable	Fixed	Variable	Fixed
${f}_{12}^{(Y)}$	< 0	< 0	< 0	< 0	< 0
${f}_{21, eff}^{(Y)}$	> 0	< 0	> 0	> 0	< 0
$\left|{f}_{21, eff}^{(Y)}\right|/\left|{f}_{12}^{(Y)}\right|$	< 1	> 1	< 1	> 1	= 1

The calculations were performed for the point-wake model^21^ of the particle interaction with the rather strong nonreciprocity $\left( {{{f_{21}^{{({\text{Y}})}} } \mathord{\left/ {\vphantom {{f_{21}^{{({\text{Y}})}} } {f_{12}^{{({\text{Y}})}} }}} \right. \kern-\nulldelimiterspace} {f_{12}^{{({\text{Y}})}} }} < 0} \right)$ taking into account the conditions of the vertical stability^21,55^ and the charge fluctuation sufficient to satisfy the condition $\left| {{{f_{21,eff}^{{({\text{Y}})}} } \mathord{\left/ {\vphantom {{f_{21,eff}^{{({\text{Y}})}} } {f_{12}^{{({\text{Y}})}} }}} \right. \kern-\nulldelimiterspace} {f_{12}^{{({\text{Y}})}} }}} \right| > 1$.

**Figure 9:**
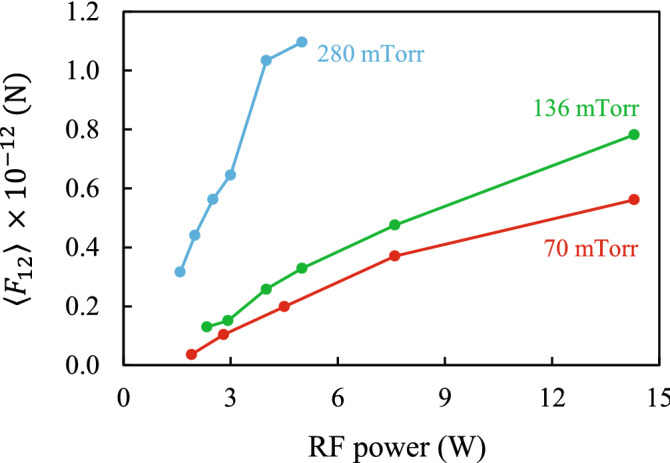
Estimation of the force $\left\langle {F_{12}^{{}} } \right\rangle$ with which the lower particle acts on the upper one.

Under these assumptions, we can also estimate the force with which the lower particle acts on the upper one. Using the approximate equation^46^

5 $$\left\langle {F_{12}^{{}} } \right\rangle \approx Mf_{12}^{{({\text{X}})}} \Delta,$$

our calculations show that for the point-wake model and the Kompaneets model with values of derivatives close to the experimental ones, Eq. (5) has an error of 5–20%. The estimation of the force $\left\langle {F_{12}^{{}} } \right\rangle$ obtained from Eq. (5) is presented in Fig. 9 which shows that this force increases with increasing power and pressure. Note that in Ref.^46^ there is also an equation (see Eq. 15) for determining the specific force $\left\langle {F_{21}^{{}} } \right\rangle$ from the balance of forces. However, this equation is not applicable here due to the lack of accuracy in determining the relationship ${{f_{1}^{{({\text{X}})}} } \mathord{\left/ {\vphantom {{f_{1}^{{({\text{X}})}} } {f_{2}^{{({\text{X}})}} }}} \right. \kern-\nulldelimiterspace} {f_{2}^{{({\text{X}})}} }}$.

## Conclusions

A new experimental method based on an analysis of the spectral density of random processes has been presented, allowing study of the nonreciprocal effective interaction forces between particles in non-equilibrium media. In contrast to previous investigations, this method does not require a special design of the experimental setup, external perturbations of the system, pre-measurements of external fields or any assumptions concerning the type of interaction.

The proposed method was used to examine the forces of the nonreciprocal effective interaction between two microparticles in a RF produced plasma sheath, depending on the buffer gas pressure (in the range from 70 to 280 mTorr) and the discharge power (from 1.6 to 14.3 W). It was found that the spectral density of oscillations for two interacting particles in the plasma sheath differs significantly from the spectrum found for a system with a reciprocal interparticle interaction. Approximation of the experimentally measured spectral densities employing analytical equations allowed a determination of the derivatives of the specific interaction forces and of the specific confinement forces, depending on the RF power and the gas pressure. Across all discharge parameters, the effective interaction between the particles was nonreciprocal. It was also observed that for a nonzero relative displacement of particles in the horizontal plane, an attractive force arises, tending to put the lower particle strictly under the upper one. Using the assumption of the charge fluctuation of the lower particle we determined that the attractor of the interparticle interaction potential is located between the particles. Finally, the force which the lower particle exerts on the upper particle was measured. This force increases with increasing RF power and gas pressure.

Analyzing the vertical oscillations of particles, we found that even small changes in the charge on the lower particle (on the order of 0.1–1%), caused by thermal motion in the wake field of the upper one can lead to a significant error in determining the effective interaction between the particles. As a result, even if there is a nonreciprocal attraction between the particles, the effective interaction between the particles in the vertical projection seems to be repulsive.

The results open novel prospects of investigations of various problems in physics of disperse systems with broken interaction symmetry, where the dynamical and structural characteristics of the system depend on the geometry of interactions between particles.

## Methods

The source of stochastic motion of a microparticle in a dissipative medium can be specified by the random process $b_{{}}^{{({\upxi })}}$ which fulfills the conditions for delta-correlated Gaussian white noise:

6 $$\left\langle {b_{{}}^{{({\upxi })}} \left( t \right)} \right\rangle = 0\;\;\;\;\;\;\;\;\;\;\left\langle {b_{{}}^{{({\upxi })}} \left( t \right)b_{{}}^{{({\upxi })}} \left( {t + \tau } \right)} \right\rangle = S^{{({\upxi })}} \delta \left( \tau \right),$$

where the angle brackets indicate the average over time and $S_{{}}^{{({\upxi })}}$ is the spectral density of the process $b_{{}}^{{({\upxi })}}$. As a result of the balance between dissipation and supply of energy from the surrounding medium to the particles, their movement can be characterized by a certain magnitude of effective kinetic temperature ${{T_{{}}^{{({\upxi })}} = S_{{}}^{{({\upxi })}} M} \mathord{\left/ {\vphantom {{T_{{}}^{{({\upxi })}} = S_{{}}^{{({\upxi })}} M} {2\nu }}} \right. \kern-\nulldelimiterspace} {2\nu }}$. Note that, in active colloids and complex plasmas the fine particles may not necessarily be in thermodynamic equilibrium with the medium^25,27,29^. However, there are a lot of experimental evidence of the applicability of the stochastic model (6) for the description of dust dynamics in gas discharges. For example, the spectral density profile of oscillations of a single dust particle in a radio-frequency produced plasma sheath is fitted well to the amplitude variation of a driven damped harmonic oscillator^56^, which is possible when the random movement of a dust particle is described by the process (6). But the simplest proof of the Brownian motion of a dust particle in a RF plasma is that Gaussian distributions fit the experimental velocity distribution functions well in both the horizontal and vertical directions^50,56,57^. Note that in this case the corresponding kinetic temperatures ($T^{{({\text{X}})}} ,T^{{({\text{Y}})}}$), which the microparticle acquires due to interaction with the anisotropic plasma sheath, do not equal for different directions ($T^{{({\text{X}})}} \ne T^{{({\text{Y}})}}$)^50,57,58^, and can substantially exceed the temperatures of neutrals, ions and even electrons^58–60^.

The nonreciprocity of the wake-mediated interparticle interaction provides an additional mechanism for converting energy of the flowing ions into the kinetic energy of a pair-particle system^16,17^. As a result, the average kinetic energy of each of the particles (in a certain direction) can be greater than ${{T_{{}}^{{({\upxi })}} } \mathord{\left/ {\vphantom {{T_{{}}^{{({\upxi })}} } 2}} \right. \kern-\nulldelimiterspace} 2}$. However, in this case, the velocity distribution functions of the particles retain the Gaussian profile^14,61^. It was also shown in Ref.^17^ that in a system of two particles with the wake-mediated interparticle interaction, the income of additional energy and its redistribution in directions can be completely described using Eqs. (1) and (6).

In Ref.^62^, an analytical expression was obtained for the spectral density of the forced oscillations of the *j*-th particle in Eq. (1), which in short form can be written in the following manner:

7 $$G_{j}^{{({\upxi })}} \left( {\upomega } \right) = \frac{{\left| {\Lambda_{k} } \right|^{2} S_{j} + \left( {f_{kj}^{{({\upxi })}} } \right)^{2} S_{k}^{{({\upxi })}} }}{{\left| {\Lambda_{k} \Lambda_{j} - f_{kj}^{{({\upxi })}} f_{jk}^{{({\upxi })}} } \right|^{2} }},$$

where $\Lambda_{j} = - {\upomega }^{2} + i{\upomega }\nu + f_{j}^{{({\upxi })}} - f_{kj}^{{({\upxi })}}$, $j \ne k$. Here we assume that in general $S_{j}^{{({\upxi })}} \ne S_{k}^{{({\upxi })}}$ and $S_{j}^{{({\text{X}})}} \ne S_{j}^{{({\text{Y}})}}$ due to possible inhomogeneity and anisotropy of the plasma sheath ($T_{j}^{{({\upxi })}} \ne T_{k}^{{({\upxi })}}$, $T_{j}^{{({\text{X}})}} \ne T_{j}^{{({\text{Y}})}}$).

Making the change of variables $\delta_{ + }^{{({\upxi })}} = \delta_{1}^{{({\upxi })}} + \delta_{2}^{{({\upxi })}}$ and $\delta_{ - }^{{({\upxi })}} = \delta_{1}^{{({\upxi })}} - \delta_{2}^{{({\upxi })}}$, the spectral densities $G_{ + }^{{({\upxi })}} \left( {\upomega } \right)$ and $G_{ - }^{{({\upxi })}} \left( {\upomega } \right)$ for $\delta_{ + }^{{({\upxi })}}$ and $\delta_{ - }^{{({\upxi })}}$, respectively, are described by the following analytic expression:

8 $$\small G_{m}^{{({\upxi })}} \left( {\upomega } \right) = \frac{{\left( {\left| {\Lambda_{n} } \right|^{2} + a_{mn}^{2} } \right)\left( {S_{1}^{{({\upxi })}} +S_{2}^{{({\upxi })}} } \right) - 2a_{mn} \left( {{\upomega }^{2} + a_{nn} } \right)\left( {S_{1}^{{({\upxi })}} - S_{2}^{{({\upxi })}} } \right)}}{{\left| {\Lambda_{m} \Lambda_{n} - a_{nm} a_{mn} } \right|^{2} }},$$

where subscripts $m$ and $n$ can take the values “+” or “–”, and $m \ne n$, $\Lambda_{m(n)} = - {\upomega }^{2} + i{\upomega }\nu - a_{mm(nn)}$, $a_{mm} = - {{\left( {f_{1}^{{({\upxi })}} + f_{2}^{{({\upxi })}} } \right)} \mathord{\left/ {\vphantom {{\left( {f_{1}^{{({\upxi })}} + f_{2}^{{({\upxi })}} } \right)} 2}} \right. \kern-\nulldelimiterspace} 2}$, $a_{nn} = f_{21}^{{({\upxi })}} + f_{12}^{{({\upxi })}} - {{\left( {f_{1}^{{({\upxi })}} + f_{2}^{{({\upxi })}} } \right)} \mathord{\left/ {\vphantom {{\left( {f_{1}^{{({\upxi })}} + f_{2}^{{({\upxi })}} } \right)} 2}} \right. \kern-\nulldelimiterspace} 2}$, $a_{mn} = f_{21}^{{({\upxi })}} - f_{12}^{{({\upxi })}} - {{\left( {f_{1}^{{({\upxi })}} - f_{2}^{{({\upxi })}} } \right)} \mathord{\left/ {\vphantom {{\left( {f_{1}^{{({\upxi })}} - f_{2}^{{({\upxi })}} } \right)} 2}} \right. \kern-\nulldelimiterspace} 2}$, $a_{nm} = - {{\left( {f_{1}^{{({\upxi })}} - f_{2}^{{({\upxi })}} } \right)} \mathord{\left/ {\vphantom {{\left( {f_{1}^{{({\upxi })}} - f_{2}^{{({\upxi })}} } \right)} 2}} \right. \kern-\nulldelimiterspace} 2}$.

Analyzing the experimental data allows us to obtain the spectral density of oscillations of each of the two strongly coupled particles ($\tilde{G}_{1}^{{{{(\upxi )}}}}$ and $\tilde{G}_{2}^{{{{(\upxi )}}}}$), as well as the spectral densities of the sum and difference of these oscillations ($\tilde{G}_{ + }^{{{{(\upxi )}}}}$ and $\tilde{G}_{ - }^{{{{(\upxi )}}}}$). Recall that the upper tilde means that the value is measured in the experiment. An approximation of the experimentally measured spectral densities using Eqs. (7) and (8) allows us to determine the derivatives of the specific interaction forces $f_{21}^{{({\upxi })}}$ and $f_{12}^{{({\upxi })}}$, the derivatives of the specific confinement forces $f_{1}^{{({\upxi })}}$ and $f_{2}^{{({\upxi })}}$, along with the friction coefficient of the particles $\nu$ and the effective temperatures $T_{1}^{{({\upxi })}}$ and $T_{2}^{{({\upxi })}}$ of the stochastic processes driving the microparticles. For example, the approximation can be carried out by minimizing the residual

9 $${{\varepsilon = }}\sum\limits_{m} {\sum\limits_{{{\tilde{\omega }}}} {\left[ {\frac{{\tilde{G}_{m}^{{{{(\upxi )}}}} \left( {{\tilde{\omega }}} \right) - G_{m}^{{{{(\upxi )}}}} \left( {{\tilde{\omega }}} \right)}}{{\tilde{G}_{m}^{{{{(\upxi )}}}} \left( {{\tilde{\omega }}} \right)}}} \right]^{2} } } ,$$

where the summation is performed over all discrete frequencies, as well as over all four spectra (i.e. for $m \equiv \,$‘1’; ‘2’; ‘+’; ‘−’). Note that in some cases it is enough to know only one of the four spectra ($\tilde{G}_{1}^{{{{(\upxi )}}}}$, $\tilde{G}_{2}^{{{{(\upxi )}}}}$, $\tilde{G}_{ + }^{{{{(\upxi )}}}}$ or $\tilde{G}_{ - }^{{{{(\upxi )}}}}$) in order to determine the parameters $f_{21}^{{({\upxi })}}$, $f_{12}^{{({\upxi })}}$, $f_{1}^{{({\upxi })}}$, $f_{2}^{{({\upxi })}}$, $T_{1}^{{({\upxi })}}$, $T_{2}^{{({\upxi })}}$ and $\nu$.

Figure 3 contains graphs of approximating functions (7) and (8) obtained by minimizing the residual ${\upvarepsilon }$ using the simplex search method^63^. Summation in Eq. (9) was performed on all discrete frequencies of the fast Fourier transform, as well as on all four spectra ($m \equiv \,$ ‘1’; ‘2’; ‘+’; ‘−’). Thus, the parameters $f_{21}^{{({\upxi })}}$, $f_{12}^{{({\upxi })}}$, $f_{1}^{{({\upxi })}}$, $f_{2}^{{({\upxi })}}$, $T_{1}^{{({\upxi })}}$, $T_{2}^{{({\upxi })}}$ and $\nu$ were determined that provide the best agreement between the analytical curves and all experimentally measured spectral densities ($\tilde{G}_{1}^{{{{(\upxi )}}}}$, $\tilde{G}_{2}^{{{{(\upxi )}}}}$, $\tilde{G}_{ + }^{{{{(\upxi )}}}}$ and $\tilde{G}_{ - }^{{{{(\upxi )}}}}$).
